# Supplementary material for: Structure of the human astrovirus capsid spike in complex with the neonatal Fc receptor
Source: Nat Commun. 2025 Nov 3;16:9621. doi: 10.1038/s41467-025-65203-2 (PMC12583548; doi:10.1038/s41467-025-65203-2)
Supplement: Supplementary file 1 — Supplementary Information [file 41467_2025_65203_MOESM1_ESM.pdf]

Supplementary Information for "Structure of the human astrovirus capsid spike in complex with the neonatal Fc receptor"

Adam Lentz[1], Sarah Lanning[2], Khurshid R. Iranpur[1], Lena Ricemeyer[3], Carlos F. Arias[4], Rebecca M. DuBois[3#]

[1]Department of Microbiology & Environmental Toxicology, University of California Santa Cruz, Santa Cruz, California, USA

[2]Department of Molecular Cell and Developmental Biology, University of California Santa Cruz, Santa Cruz, California, USA

[3]Department of Biomolecular Engineering, University of California Santa Cruz, Santa Cruz, California, USA

[4]Departamento de Genética del Desarrollo y Fisiología Molecular, Instituto de Biotecnología, Universidad Nacional Autónoma de México, Cuernavaca, Morelos, Mexico

[#]Address correspondence to Rebecca M. DuBois, rmdubois@ucsc.edu

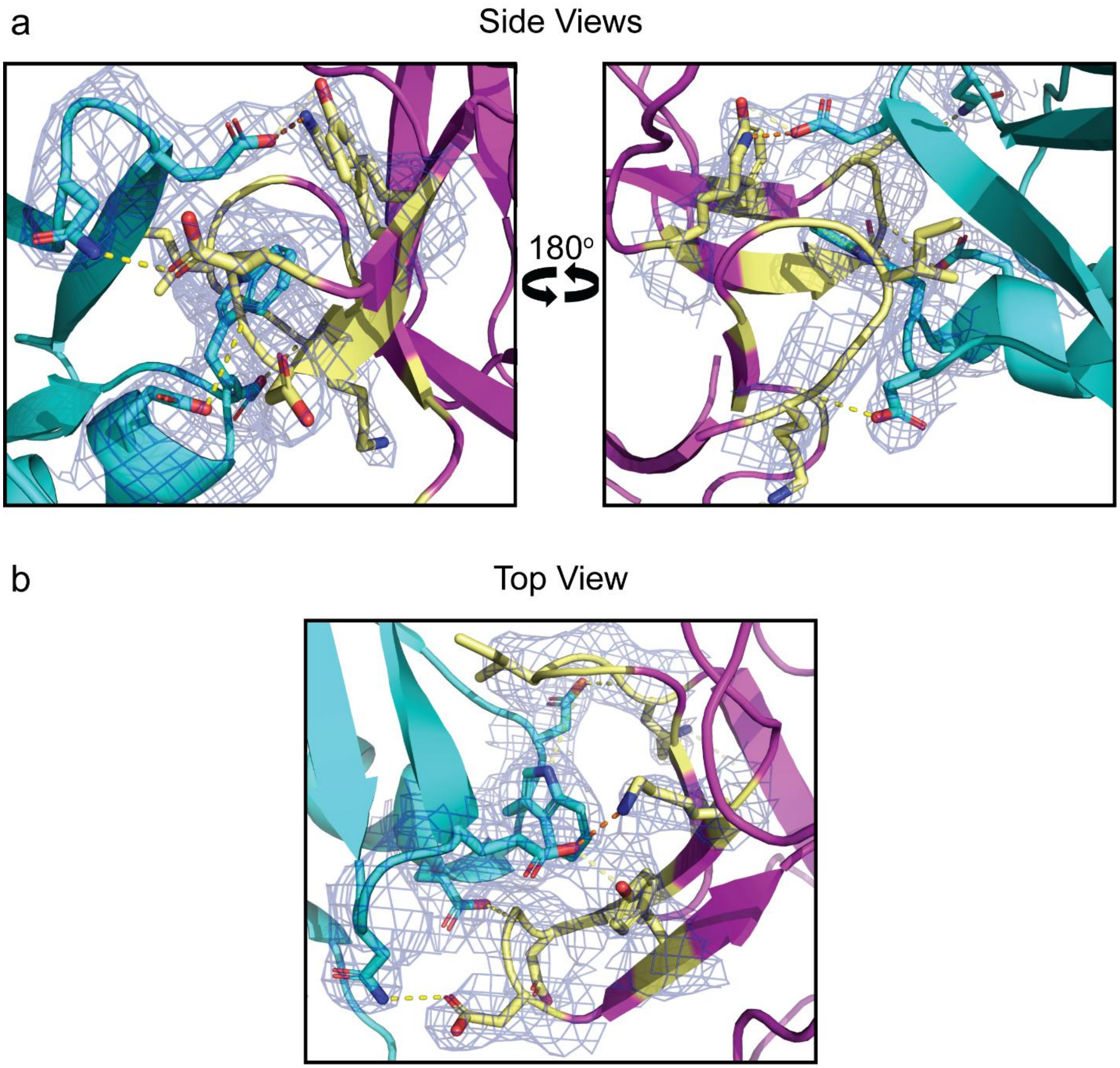

Supplementary Fig 1. Electron density at the FcRn-HAstV1 spike interface. HAstV1 spike shown in magenta and FcRn shown in teal (αFcRn). Interfacing residues on HAstV1 spike are shown in pale yellow and side chains of important residues on both proteins are shown as sticks. Hydrogen bonds shown as yellow dashed lines and electrostatic interactions shown as orange dashed lines. Electron density is shown as blue mesh. **a,** Side views of the FcRn-HAstV1 spike interface. **b,** Top view of the FcRn-HAstV1 spike interface. Source data are provided as a source data file.

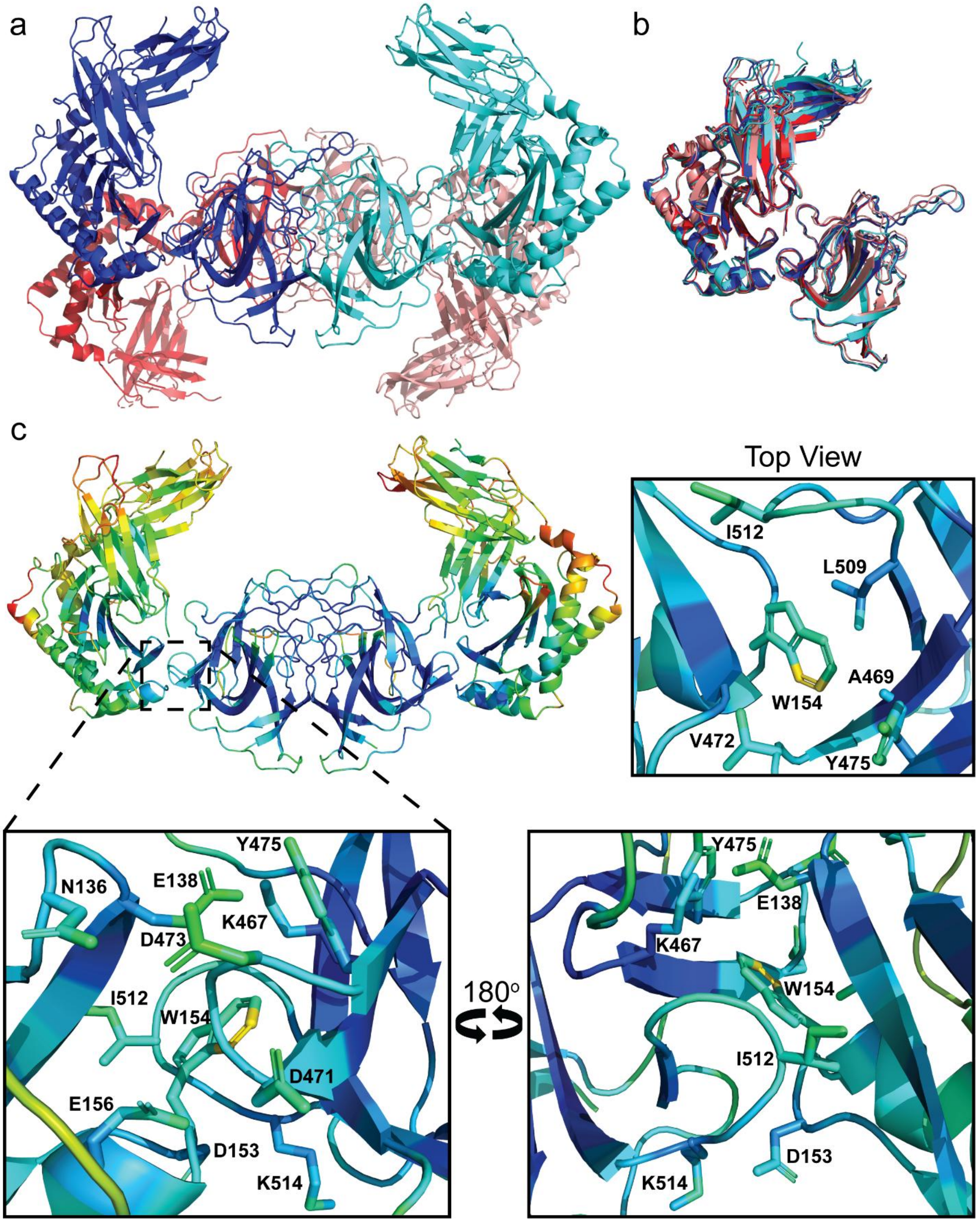

Supplementary Fig 2. The most dynamic regions in the FcRn – HAstV1 spike crystal structure are the membrane proximal regions of FcRn. a, Global view of the crystallographic

asymmetric unit containing two FcRn – HAstV1 spike complexes. Individual FcRn – HAstV1 spike half-complexes colored blue, cyan, red, and salmon respectively. **b,** Each individual FcRn – HAstV1 spike half-complex is aligned to highlight dynamic regions. All four half-complexes are very similar (RMSD 0.3-1.2 Å). **c,** One FcRn – HAstV1 spike complex colored by crystallographic B-factor from blue to red with the defined range of values from 70-180. Zoom panels show equivalent views of the binding interface as those in main Fig. 2, colored by crystallographic B-factor to depict the relative dynamic range of individual residues at the binding interface. Source data are provided as a source data file.

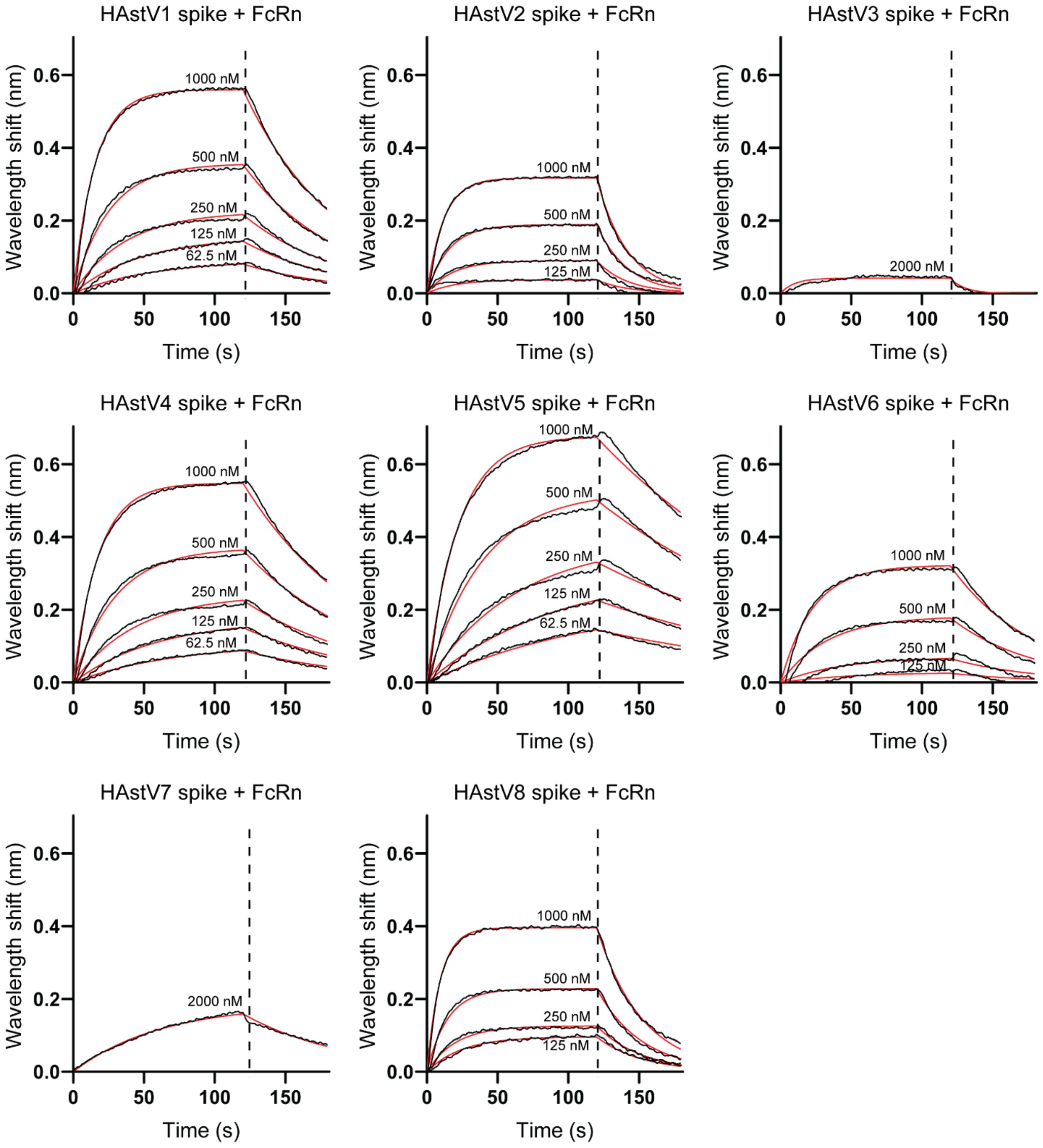

Supplementary Fig 3. HAstV1-8 spikes bind FcRn with varying affinity. Representative BLI traces for HAstV1-8 spikes binding FcRn at pH 7.0. After loading an anti-His biosensor with the respective HAstV spike protein, only association and dissociation steps are shown. A black dashed line denotes the switch between the two steps. BLI signal shown as solid black lines

and curve fits shown as solid red lines. Concentration of FcRn labeled next to each trace. Source data are provided as a source data file.

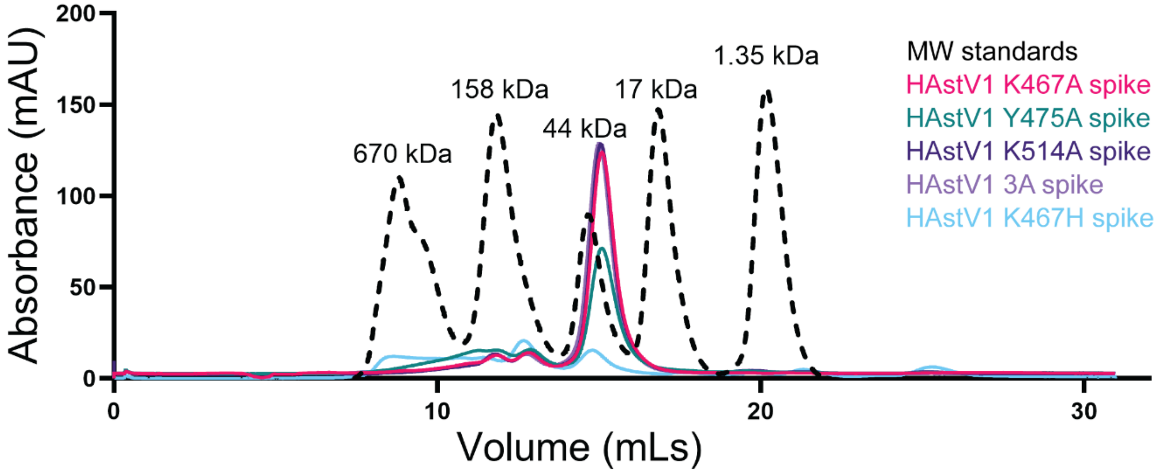

Supplementary Fig 4. HAstV1 spike mutants form dimers in solution. Size exclusion chromatography traces from HAstV1 spike mutants K467A (pink), Y475A (green), K514A (dark purple), 3A (light purple), K467H (light blue) overlayed with BioRad gel filtration standards (black dashed line). Source data are provided as a source data file.

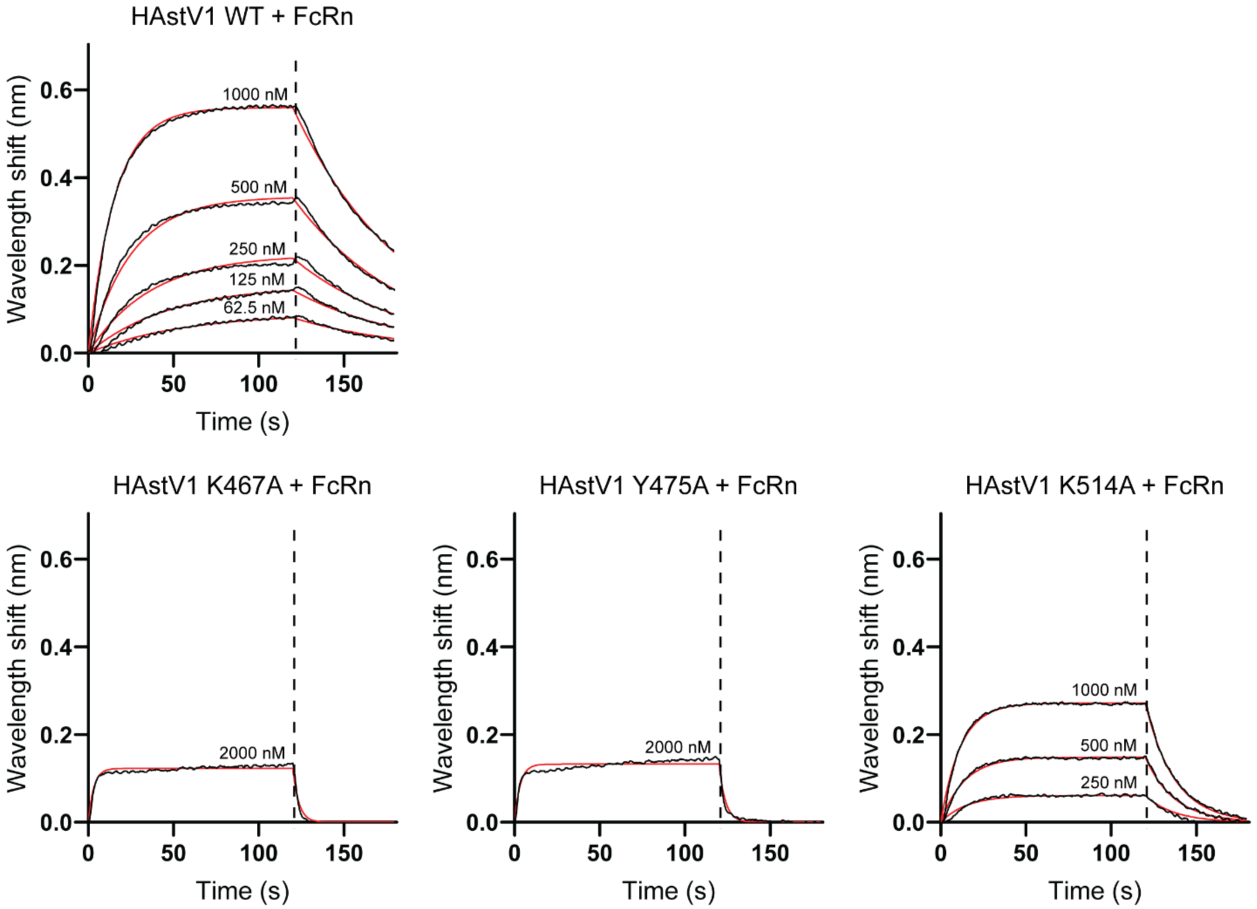

Supplementary Fig 5. HAstV1 spike mutants K467A, Y475A, and K514A show reduced binding to FcRn. Representative BLI traces for HAstV1 spike mutants binding to FcRn at pH 7.0. After loading an anti-His biosensor with the respective HAstV spike protein, only association and dissociation steps are shown. A black dashed line denotes the switch between the two steps. BLI signal shown as solid black lines and curve fits shown as solid red lines. Concentration of FcRn labeled next to each trace. Source data are provided as a source data file.

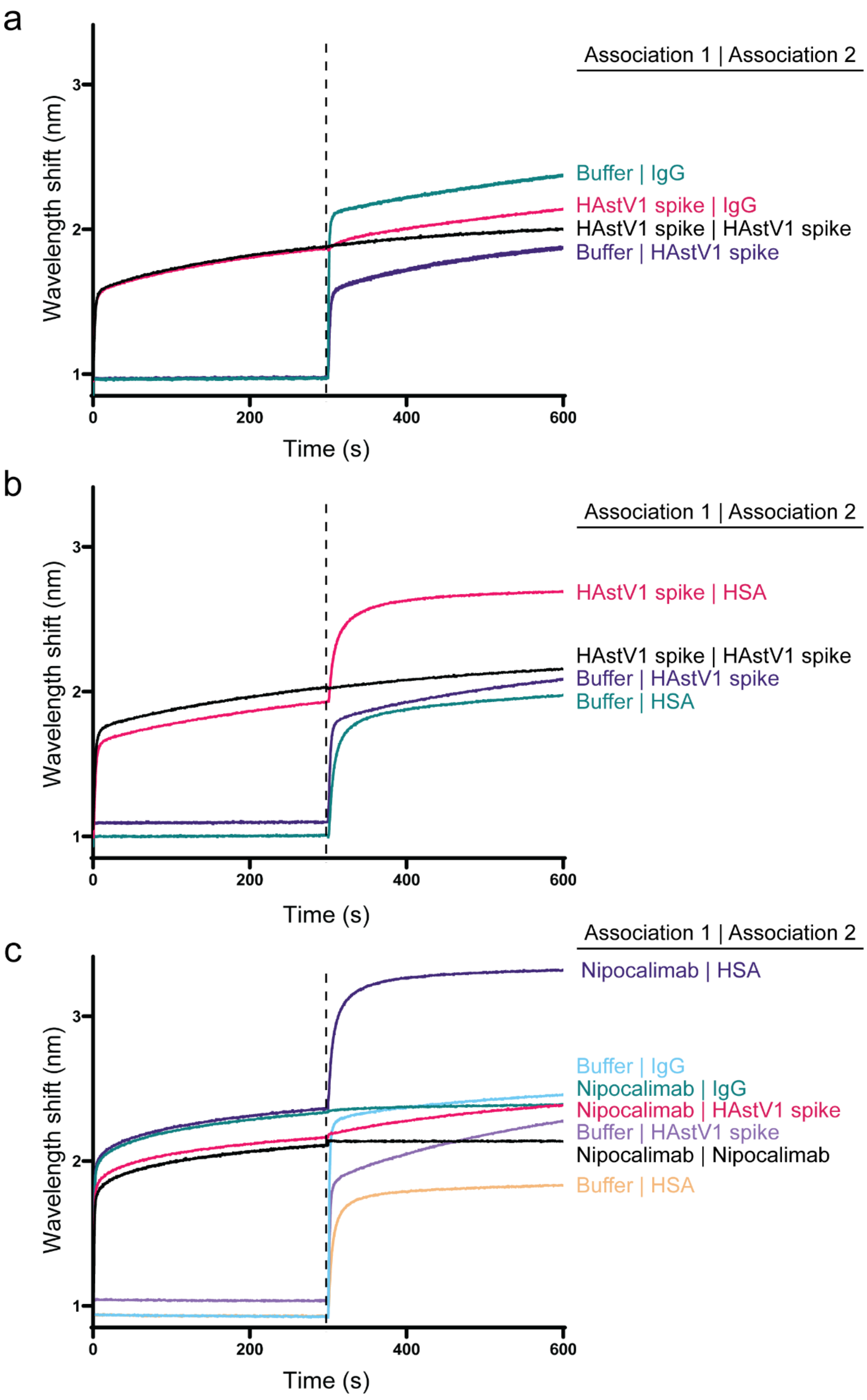
a
Association 1 | Association 2
Buffer | IgG
HAstV1 spike | IgG
HAstV1 spike | HAstV1 spike
Buffer | HAstV1 spike
Wavelength shift (nm)
Time (s)
b
Association 1 | Association 2
HAstV1 spike | HSA
HAstV1 spike | HAstV1 spike
Buffer | HAstV1 spike
Buffer | HSA
Wavelength shift (nm)
Time (s)
c
Association 1 | Association 2
Nipocalimab | HSA
Buffer | IgG
Nipocalimab | IgG
Nipocalimab | HAstV1 spike
Buffer | HAstV1 spike
Nipocalimab | Nipocalimab
Buffer | HSA
Wavelength shift (nm)
Time (s)

Supplementary Fig 6. HAstV1 spike competes with IgG for binding to FcRn and nipocalimab blocks HAstV1 spike binding to FcRn Representative BLI traces for each HAstV1 spike competition assay with FcRn's other binding partners, IgG (**a**) and HSA (**b**), and the FcRn antibody, nipocalimab (**c**), at pH 5.0. Only association steps are shown. The black dashed line marks the switch between the two association steps. Association 1 saturates the FcRn loaded biosensor with HAstV1 spike (**a,b**), nipocalimab (**c**), or contains only buffer for the control biosensors. Association 2 shows the binding signal from the respective FcRn binding protein. Source data are provided as a source data file.

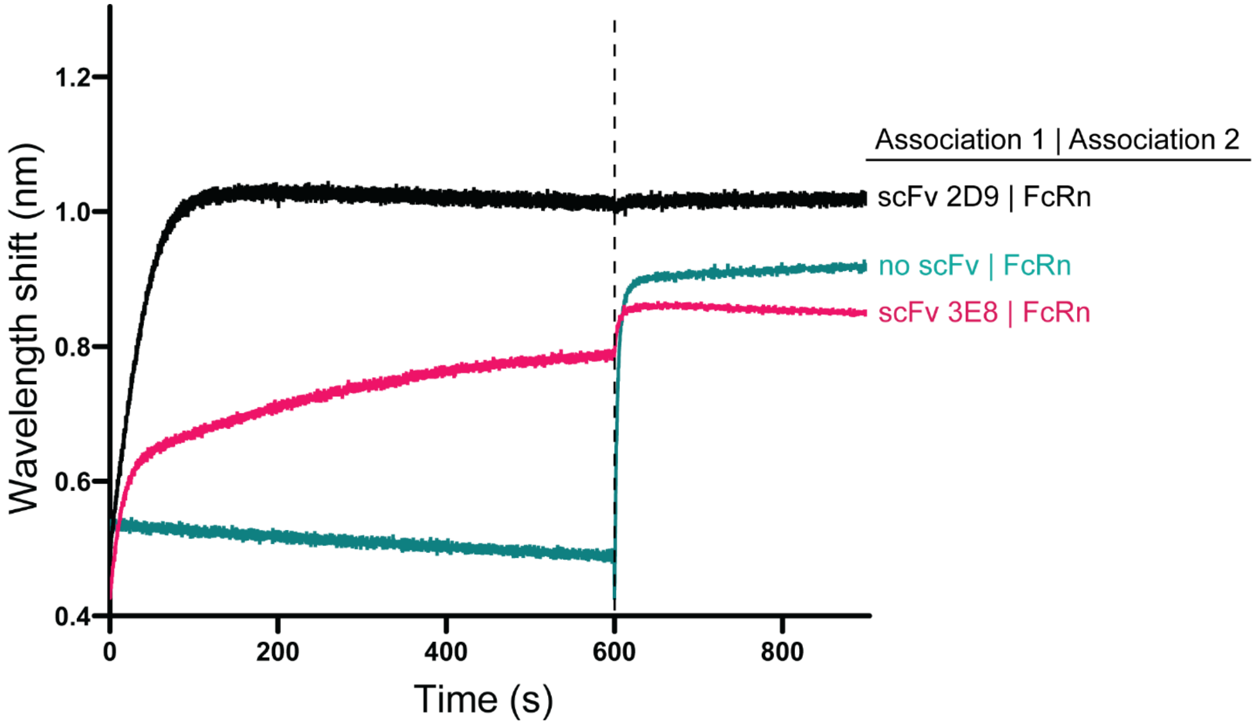

Supplementary Fig 7. Both scFv 2D9 and scFv 3E8 block FcRn binding to HAstV spike. Representative BLI traces for each FcRn competition with scFv 2D9 and scFv 3E8 at pH 7.0. Only association steps are shown. The black dashed line denotes the split between the two association steps. Association 1 pre-complexes an HAstV spike loaded biosensor with the respective scFv or no scFv. Association 2 shows the binding signal of FcRn to the respective complex. Source data are provided as a source data file.

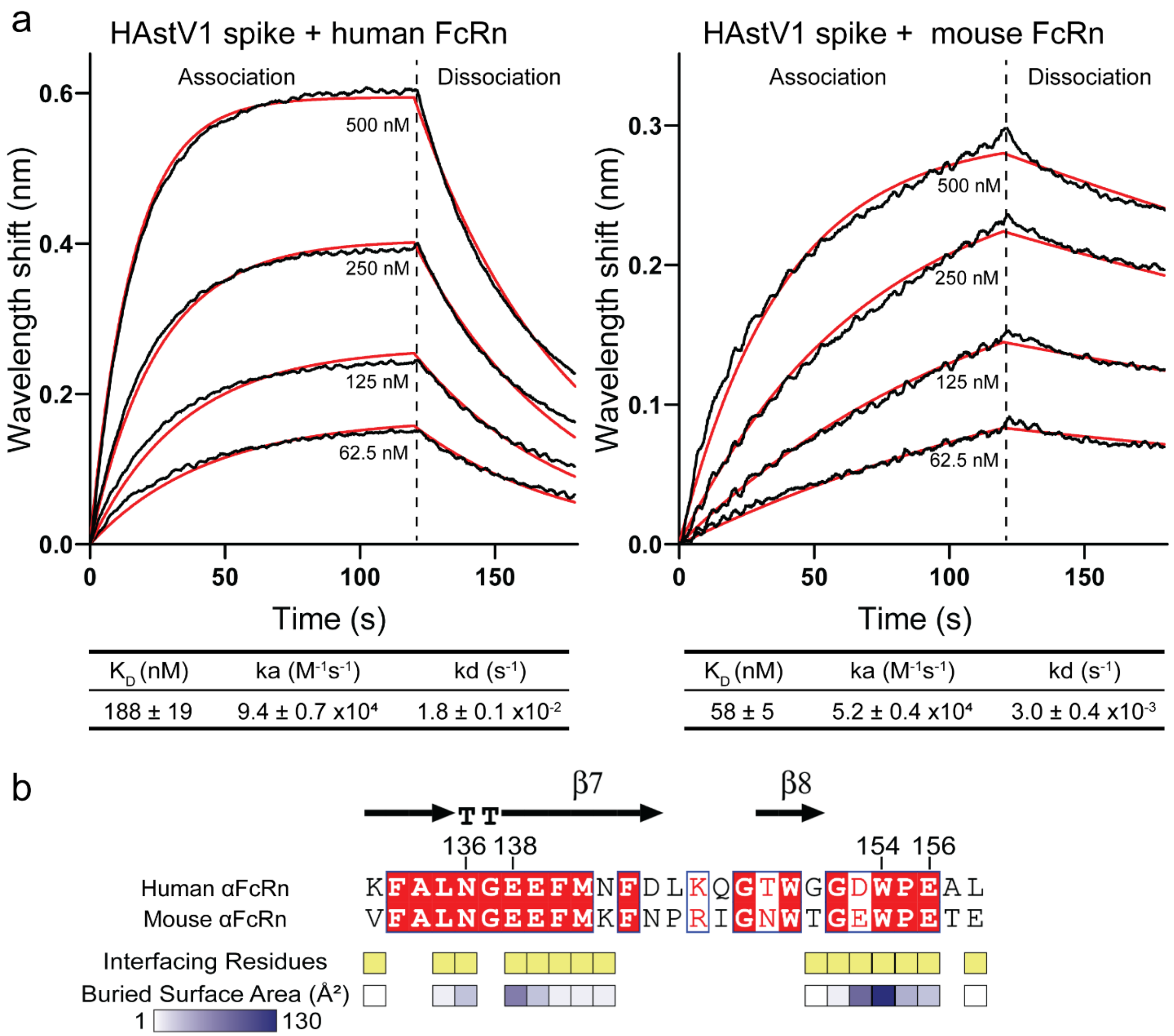

Supplementary Fig 8. HAstV1 spike binds to both human and mouse FcRn. a, Representative BLI traces for HAstV1 spike binding to human (left) and mouse (right) FcRn at pH 7.0. Only association and dissociation steps are shown. The black dashed line denotes the switch between the two steps. Concentrations of human or mouse FcRn are shown above each trace. Binding equilibrium constant (K_D), association rate (ka), and dissociation rate (kd) are reported as an average of three replicates ± standard deviation. **b,** Alignment of the interfacing region of αFcRn made using the Clustal Omega and ESPript servers. Interfacing residues are marked with a pale-yellow box below the alignment. Buried surface area determined with

PDBePISA and shown as a box colored with a white to blue gradient below the alignment. Important conserved residues are numbered based on human αFcRn above the alignment. Secondary structure information from human αFcRn shown above the alignment. Source data are provided as a source data file.

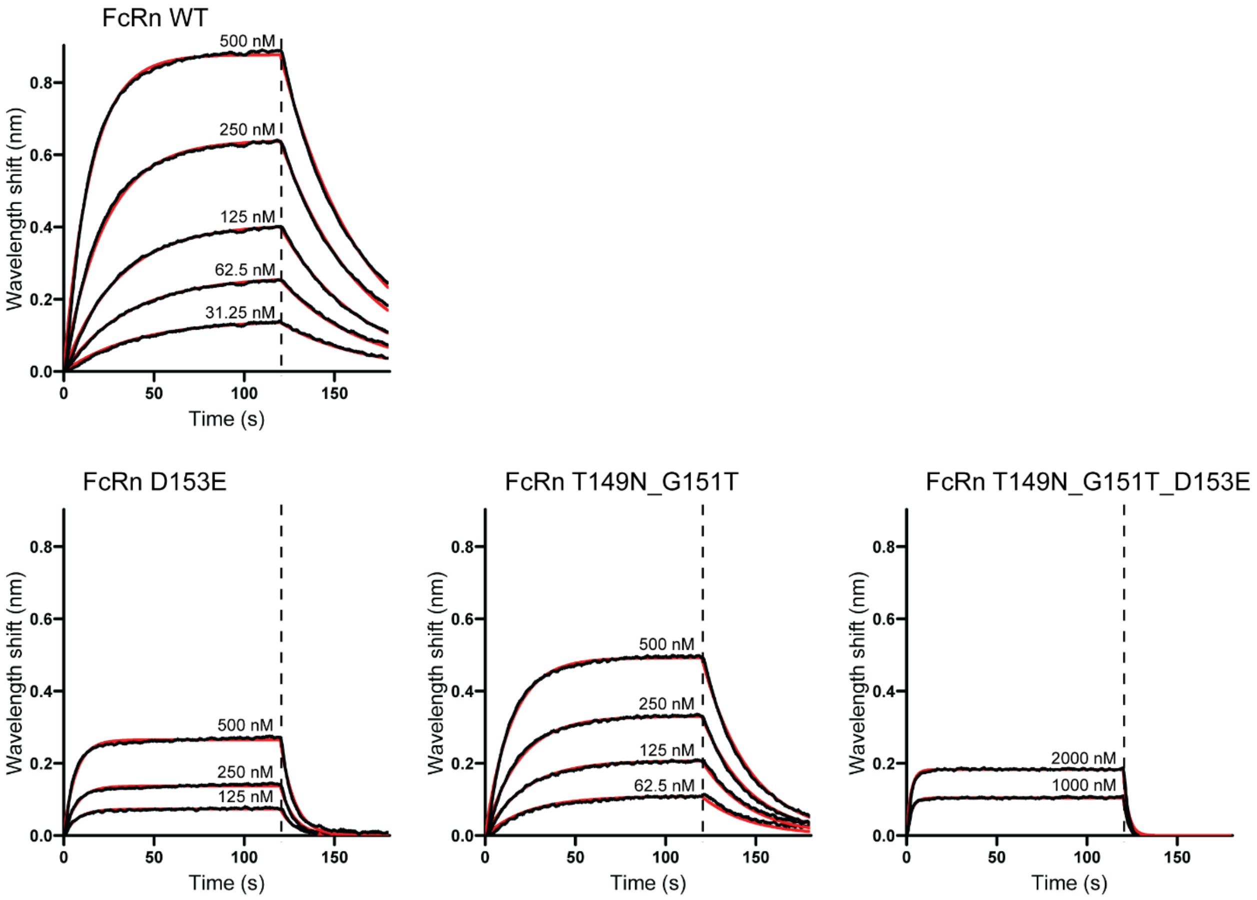

Supplementary Fig 9. Human FcRn mutants mimicking the mouse FcRn binding interface show reduced binding to HAstV1 spike. Representative BLI traces for FcRn mutants binding to HAstV1 spike at pH 7.0. After loading a streptavidin biosensor with biotinylated HAstV1 spike, only association and dissociation steps are shown. A black dashed line denotes the switch between the two steps. BLI signal shown as solid black lines and curve fits shown as solid red lines. Concentration of respective wild-type or mutant FcRn labeled next to each trace. Source data are provided as a source data file.

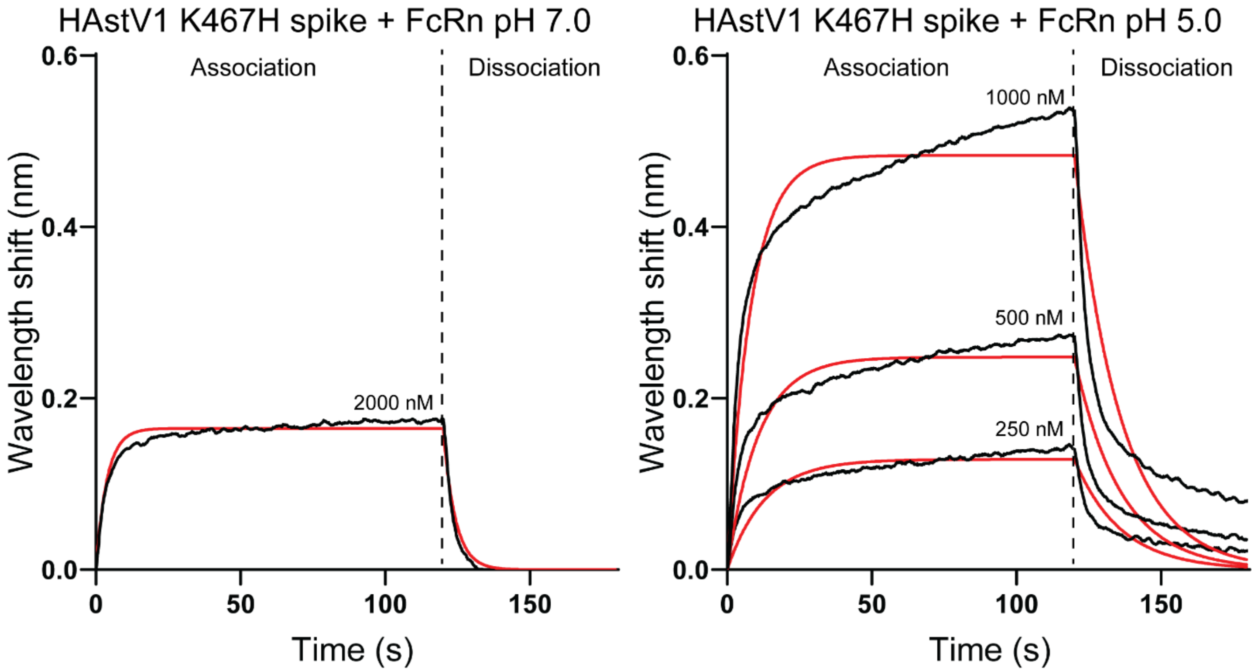

K_D (μM)	ka ($M^{-1}s^{-1}$)	kd (s^{-1})
22.5 ± 3.2	1.1 ± 0.2 x10^4	2.4 ± 0.1 x10^{-1}

K_D (μM)	ka ($M^{-1}s^{-1}$)	kd (s^{-1})
0.8 ± 0.2	6.6 ± 0.6 x10^4	5.1 ± 1.3 x10^{-2}

Supplementary Fig 10. HAstV1 K467H spike mutant has improved binding to FcRn at pH 5.0 compared to pH 7.0. Representative BLI traces of streptavidin biosensors loaded with biotinylated HAstV1 K467H spike and dipped into FcRn at pH 7.0 (left) or pH 5.0 (right). Concentrations of FcRn used are labeled next to each trace. BLI binding signals are shown as black lines and curve fits are shown as red lines. Binding equilibrium constant (K_D), association rate (ka), and dissociation rate (kd) are reported as an average of three replicates ± standard deviation. Source data are provided as a source data file.

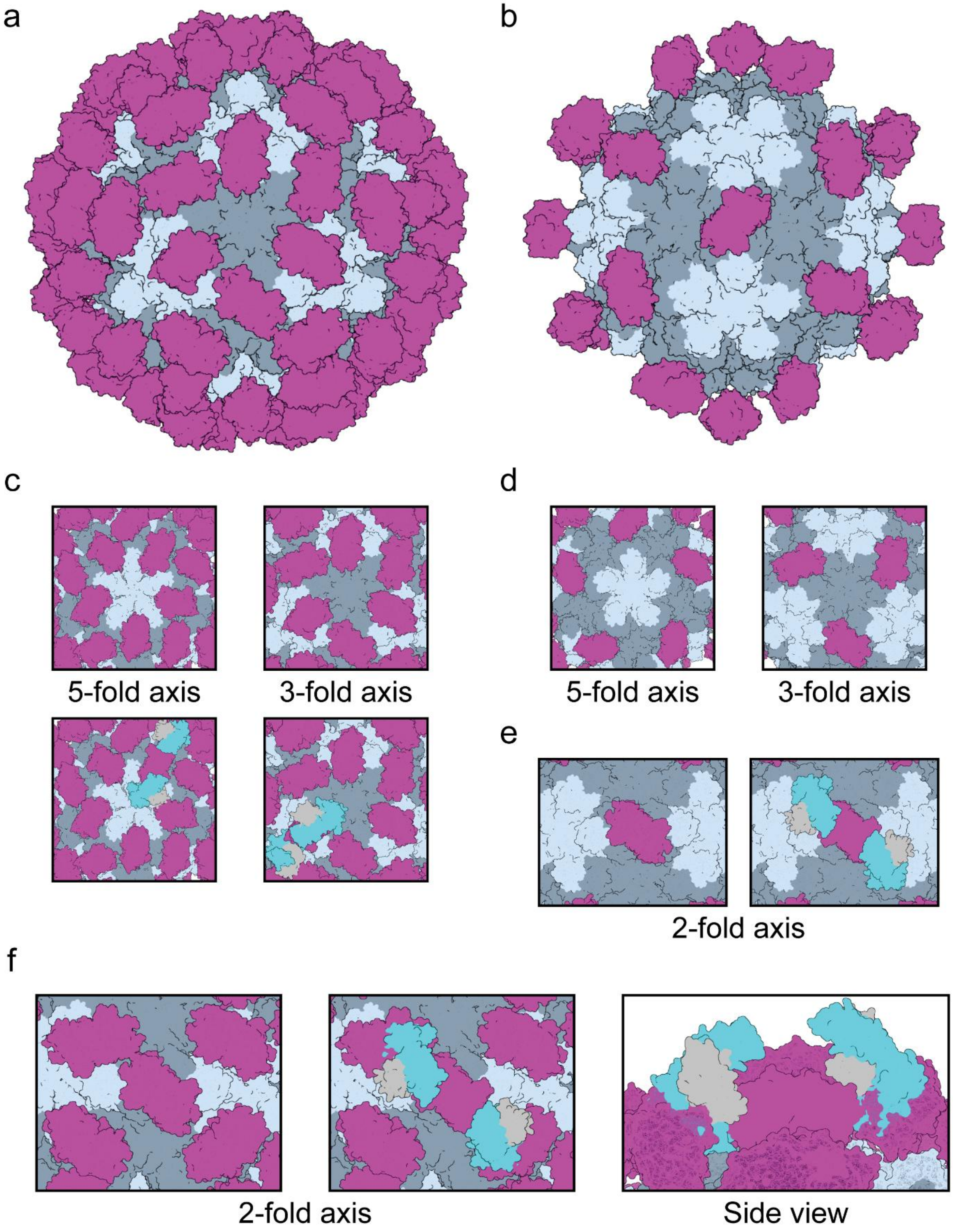

Supplementary Fig 11. Model of FcRn binding to the immature and mature HAstV virion.

a, Model of the immature HAstV1 virion. **b,** Model of the mature HAstV1 virion. **c,** Views of the

5-fold and 3-fold symmetry axes on the immature HAstV1 virion without FcRn (top) and with FcRn (bottom). **d,** Views of the 5-fold and 3-fold symmetry axes on the mature HAstV1 virion. **e,** Views of the 2-fold symmetry axis on the mature HAstV1 virion without FcRn (left) and with FcRn (right). **f,** Views of the 2-fold symmetry axis on the immature HAstV1 virion without FcRn (left) and with FcRn from the top (middle) and side (right). Image made using ChimeraX (v1.8)

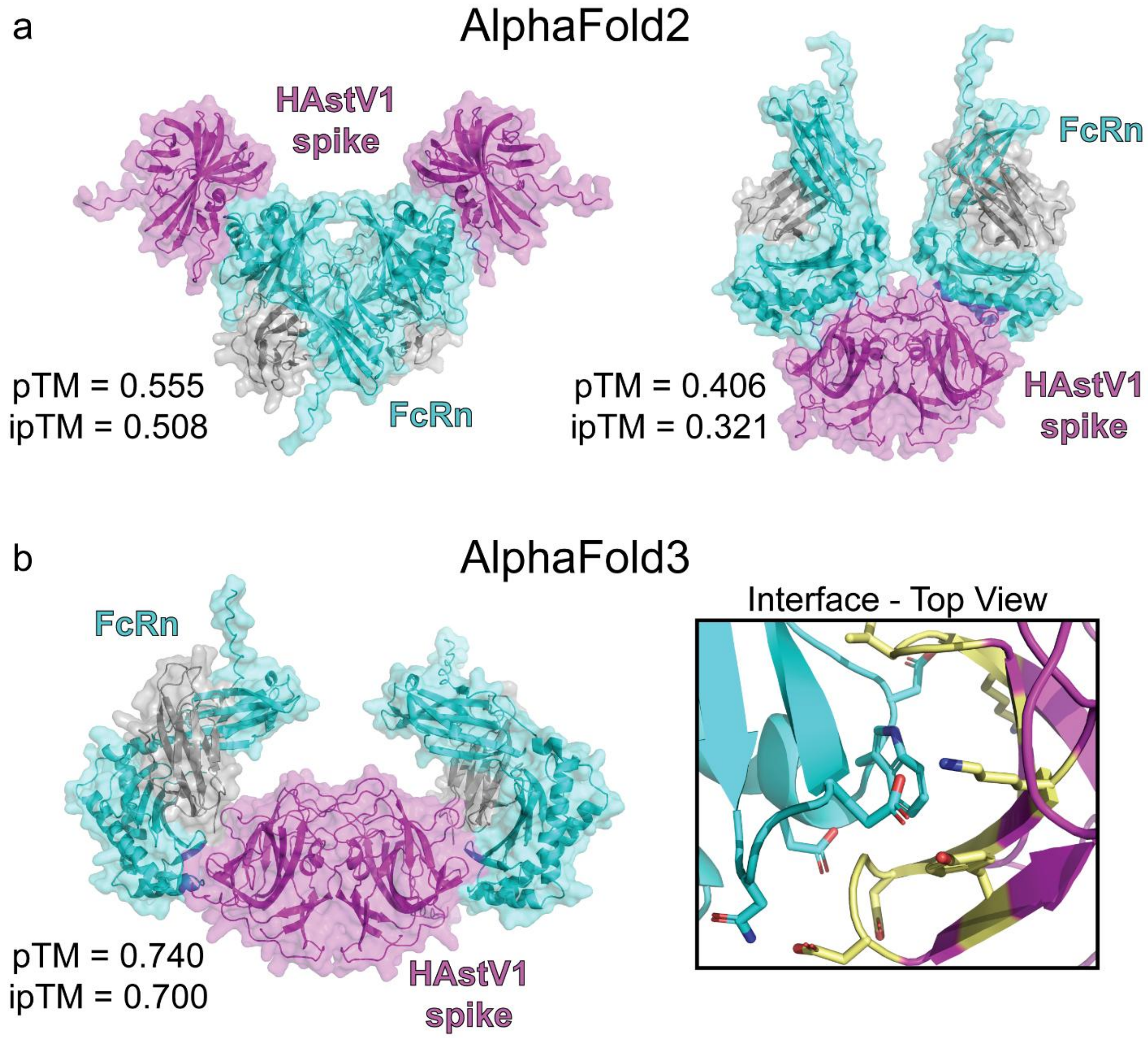

Supplementary Fig 12. AlphaFold3 correctly predicted the structure of HAstV1 spike bound to FcRn. HAstV1 spike shown in magenta and FcRn shown in teal (αFcRn) and grey (β2M). **a,** The two highest confidence AlphaFold2 structure predictions for HAstV1 spike bound

to FcRn. **b,** The highest confidence AlphaFold3 structure prediction for HAstV1 spike bound to FcRn (left) and a zoom in of the interface between the two proteins (right). pTM and ipTM scores provided to display the confidence in the accuracy of the predicted structure overall (pTM) and at the interface (ipTM).

Supplementary Table 1. Data collection and refinement statistics (molecular replacement).

	HAstV1 spike – FcRn (PDB: 9DBT)
**Data collection**	
Space group	$P2\ 2_1\ 2_1$
Cell dimensions	
a, *b*, *c* (Å)	87.00, 152.99, 255.36
α, β, γ (°)	90.00, 90.00, 90.00
Resolution (Å)	255.36-3.40 (3.46-3.40)*
R_{merge}	0.169 (1.094)
Rpim	0.039 (0.249)
$I / \sigma I$	9.4 (0.9)
$CC_{1/2}$	0.998 (0.854)
Completeness (%)	100.0 (100.0)
Redundancy	19.7 (20.2)
Resolution (Å) where $I / \sigma I \geq 2.0$	3.83
Wilson B factor	94.916
**Refinement**	
Resolution (Å)	98.03-3.40 (3.52-3.40)
No. reflections	47,769 (4,704)
R_{work} / R_{free}	0.209 / 0.263 (0.321 / 0.360)
No. atoms	
Protein	17,935

Chains A, C, F, and I (αFcRn)	1,709-2,097
Chains B, D, G, and J (β2M)	738-829
Chains E, H, K, and L (HAstV1 spike)	1,687
Ligand/ion	
Water	
B-factors	
Protein	112.69
Chains A, C, F, and I (αFcRn)	117.02-127.17
Chains B, D, G, and J (β2M)	119.75-149.99
Chains E, H, K, and L (HAstV1 spike)	85.91-102.80
Ligand/ion	
Water	
R.m.s. deviations	
Bond lengths (Å)	0.011
Bond angles (°)	1.29

*Values in parentheses are for highest-resolution shell.

Supplementary Table 2. Binding affinities of mutant FcRn to HAstV1 spike at pH 7.0.

Mutant	K_D (μM)	ka ($M^{-1}s^{-1}$)	kd (s^{-1})	X^2	R^2
WT	0.23 ± 0.01	9.3 ± 0.3 $x10^4$	2.23 ± 0.05 $x10^{-2}$	0.179	0.999
D153E	1.6 ± 0.7	8.5 ± 2.6 $x10^4$	1.4 ± 0.3 $x10^{-1}$	0.144	0.993
T149N_G151T	0.5 ± 0.1	7.4 ± 0.9 $x10^4$	3.4 ± 0.3 $x10^{-2}$	0.162	0.998
*T149N_G151T_D153E	11.9 ± 0.9	3.1 ± 0.1 $x10^4$	3.7 ± 0.2 $x10^{-1}$	0.307	0.974

*Kinetics estimated from two FcRn concentrations (2 μM and 1 μM).
