# Supplementary material for: Structure of the human astrovirus capsid spike in complex with the neonatal Fc receptor
Source: Nat Commun. 2025 Nov 3;16:9621. doi: 10.1038/s41467-025-65203-2 (PMC12583548; doi:10.1038/s41467-025-65203-2)
Supplement: Supplementary file 2 — Reporting summary [file 41467_2025_65203_MOESM2_ESM.pdf]

nature portfolio

Corresponding author(s): Rebecca M. DuBois

Last updated by author(s): Oct 2, 2025

Reporting Summary

Nature Portfolio wishes to improve the reproducibility of the work that we publish. This form provides structure for consistency and transparency in reporting. For further information on Nature Portfolio policies, see our Editorial Policies and the Editorial Policy Checklist.

Statistics

For all statistical analyses, confirm that the following items are present in the figure legend, table legend, main text, or Methods section.

n/a	Confirmed	
☐	☒	The exact sample size (*n*) for each experimental group/condition, given as a discrete number and unit of measurement
☐	☒	A statement on whether measurements were taken from distinct samples or whether the same sample was measured repeatedly
☐	☒	The statistical test(s) used AND whether they are one- or two-sided<br>*Only common tests should be described solely by name; describe more complex techniques in the Methods section.*
☒	☐	A description of all covariates tested
☒	☐	A description of any assumptions or corrections, such as tests of normality and adjustment for multiple comparisons
☐	☒	A full description of the statistical parameters including central tendency (e.g. means) or other basic estimates (e.g. regression coefficient) AND variation (e.g. standard deviation) or associated estimates of uncertainty (e.g. confidence intervals)
☐	☒	For null hypothesis testing, the test statistic (e.g. *F*, *t*, *r*) with confidence intervals, effect sizes, degrees of freedom and *P* value noted<br>*Give P values as exact values whenever suitable.*
☒	☐	For Bayesian analysis, information on the choice of priors and Markov chain Monte Carlo settings
☒	☐	For hierarchical and complex designs, identification of the appropriate level for tests and full reporting of outcomes
☒	☐	Estimates of effect sizes (e.g. Cohen's *d*, Pearson's *r*), indicating how they were calculated

Our web collection on statistics for biologists contains articles on many of the points above.

Software and code

Policy information about availability of computer code

Data collection	Crystal x-ray diffraction data were collected using beam line 501 at The Advanced Light Source. Biolayer Interferometry data was collected using an Octet RED384 with the Data Acquisition Software v.11.1.1.19. ELISA absorbance data was collected using a Molecular Devices SPECTRAmax PLUS plate reader with SoftMax Pro v4.8.
Data analysis	Crystal data were processed using DIALS v.3.18, COOT v.9.8.92, PHENIX v.1.20.1-4487, and PyMOL v.3.1.4.1. Biolayer Interferometry data was analyzed using an Octet RED384 with the Data Analysis HT Software v7. Graphs generated with GraphPad Prism v.10.6.1.

For manuscripts utilizing custom algorithms or software that are central to the research but not yet described in published literature, software must be made available to editors and reviewers. We strongly encourage code deposition in a community repository (e.g. GitHub). See the Nature Portfolio guidelines for submitting code & software for further information.

Data

Policy information about availability of data

All manuscripts must include a data availability statement. This statement should provide the following information, where applicable:

- Accession codes, unique identifiers, or web links for publicly available datasets
- A description of any restrictions on data availability
- For clinical datasets or third party data, please ensure that the statement adheres to our policy

Source data are provided with this paper. The coordinates and structure factors for the FcRn – HAstV1 spike complex structure have been deposited in the Protein

Data Bank under accession code 9DBT [https://doi.org/10.2210/pdb9DBT/pdb]. The raw diffraction data has been made available at the Integrated Resource for Reproducibility in Macromolecular Crystallography 2.0 [https://www.proteindiffraction.org/project/data_9DBT/].

Research involving human participants, their data, or biological material

Policy information about studies with human participants or human data. See also policy information about sex, gender (identity/presentation), and sexual orientation and race, ethnicity and racism.

Reporting on sex and gender	Sex and gender were not considered in study design.
Reporting on race, ethnicity, or other socially relevant groupings	Race, ethnicity, or other socially relevant groupings were not considered in study design.
Population characteristics	No human research participants were included in the study.
Recruitment	see above
Ethics oversight	see above

Note that full information on the approval of the study protocol must also be provided in the manuscript.

Field-specific reporting

Please select the one below that is the best fit for your research. If you are not sure, read the appropriate sections before making your selection.

☒ Life sciences ☐ Behavioural & social sciences ☐ Ecological, evolutionary & environmental sciences

For a reference copy of the document with all sections, see nature.com/documents/nr-reporting-summary-flat.pdf

Life sciences study design

All studies must disclose on these points even when the disclosure is negative.

Sample size	Only in vitro assays on isolated macromolecules were performed, rendering sample size calculations unnecessary. Assays were performed in triplicate to ensure replication of the results.
Data exclusions	Outliers and weak/low quality data obtained from X-ray diffraction were excluded according to standard practices. Some data from biolayer interferometry experiments were excluded as outliers based on instrument limits of detection and/or poor fit to the kinetic model.
Replication	Binding experiments were performed in triplicate and successfully replicated.
Randomization	Not relevant to study--In vitro binding assays were performed across all experimental groups.
Blinding	Not relevant to study--no randomization of group allocation.

Reporting for specific materials, systems and methods

We require information from authors about some types of materials, experimental systems and methods used in many studies. Here, indicate whether each material, system or method listed is relevant to your study. If you are not sure if a list item applies to your research, read the appropriate section before selecting a response.

Materials & experimental systems

n/a	Involved in the study	
☐	☒	Antibodies
☐	☒	Eukaryotic cell lines
☒	☐	Palaeontology and archaeology
☒	☐	Animals and other organisms
☒	☐	Clinical data
☒	☐	Dual use research of concern
☒	☐	Plants

Methods

n/a	Involved in the study	
☒	☐	ChIP-seq
☒	☐	Flow cytometry
☒	☐	MRI-based neuroimaging

Antibodies

Antibodies used	Recombinant antibodies 2D9, 3E8, 4B6, 3B4, and 3H4 were generated in-house. Nipocalimab was purchased from MedChemExpress (Catalog # HY-P99037, lot # 708910).
Validation	Reactivity of antibodies 2D9 and 3E8 are demonstrated in Ricemeyer et al. 2022 (DOI: https://doi.org/10.1128/JVI.01415-21) and in this study (Fig. 4 and Supplementary Fig. 5). Reactivity of antibodies 4B6, 3B4, and 3H4 are demonstrated in Lanning et al. 2025 (DOI: https://doi.org/10.1128/jvi.01619-24).

Eukaryotic cell lines

Policy information about cell lines and Sex and Gender in Research

Cell line source(s)	CHO-S cells sourced from Invitrogen (514448).
Authentication	None of the cell lines used were authenticated.
Mycoplasma contamination	Cell lines were not tested for Mycoplasma contamination.
Commonly misidentified lines (See ICLAC register)	*Name any commonly misidentified cell lines used in the study and provide a rationale for their use.*

Plants

Seed stocks	*Report on the source of all seed stocks or other plant material used. If applicable, state the seed stock centre and catalogue number. If plant specimens were collected from the field, describe the collection location, date and sampling procedures.*
Novel plant genotypes	*Describe the methods by which all novel plant genotypes were produced. This includes those generated by transgenic approaches, gene editing, chemical/radiation-based mutagenesis and hybridization. For transgenic lines, describe the transformation method, the number of independent lines analyzed and the generation upon which experiments were performed. For gene-edited lines, describe the editor used, the endogenous sequence targeted for editing, the targeting guide RNA sequence (if applicable) and how the editor was applied.*
Authentication	*Describe any authentication procedures for each seed stock used or novel genotype generated. Describe any experiments used to assess the effect of a mutation and, where applicable, how potential secondary effects (e.g. second site T-DNA insertions, mosiacism, off-target gene editing) were examined.*
